# The (Female) Graduate: Choice and Consequences of Women’s Clothing

**DOI:** 10.3389/fpsyg.2018.02401

**Published:** 2018-11-29

**Authors:** Fabio Fasoli, Anne Maass, Chiara Volpato, Maria Giuseppina Pacilli

**Affiliations:** ^1^School of Psychology, University of Surrey, Guildford, United Kingdom; ^2^Instituto Universitário de Lisboa (ISCTE-IUL), CIS-IUL, Lisbon, Portugal; ^3^Dipartimento di Psicologia dello Sviluppo e della Socializzazione, Università di Padova, Padua, Italy; ^4^Facoltà di Psicologia, Università degli Studi di Milano-Bicocca, Milan, Italy; ^5^Dipartimento di Scienze Politiche, Università degli Studi di Perugia, Perugia, Italy

**Keywords:** graduation, outfit, clothing, sexual objectification, television

## Abstract

This research investigates how female students choose their graduation outfit and how clothing affects observers’ judgments. In Study 1, we manipulated the students’ graduation outfit so as to look professional or sexy. Female peers, adults, and professors formed a first impression about the students, their thesis work and guessed their graduation scores (thesis points and final mark). All participant groups judged the professionally dressed students as more competent, as having put more effort in their thesis, and as having obtained better scores than when the same students dressed sexy. In Studies 2 and 3 we replicated previous findings by using photos portraying real students in their actual graduation outfits. We found that sexy clothing, considered inappropriate for the occasion, affected estimated and actual graduation scores negatively and that this effect was mediated by perceived incompetence. Results are discussed with respect to women’s evaluation on the basis of their appearance.

## Introduction

In Western societies it is common and culturally legitimate to consider women’s value in relation to the pleasantness of their appearance (Bartky, 1990; Loughnan and Pacilli, 2014). Clothing choices are a crucial part of this appearance. Also, the physical attractiveness of women is increasingly equated with sexiness (Allen and Gervais, 2012). As a consequence, women tend to mold their public self-image through a sexualized appearance (Ward et al., 2016).

Often, this sexualized appearance is required in an explicit way, such as when famous women were banned from the red carpet in Cannes or receptionists were sent home because they were not wearing high heels and, thus, were not conforming to the “dress code” and norms associated to this specific context (Barnes, 2015; Johnston, 2016). At other times, sexualized outfits are considered inappropriate. For instance, female students were punished, or even banned from college, because their skirts were judged too short or their tops and pants too tight for the school context (Valenti, 2015). Thus, women constantly need to monitor their looks and modify their outfits depending on what is required in a given setting (Smolak et al., 2014). Whether an outfit is too sexy for certain contexts and whether a woman should or should not wear a given outfit is a question of debate. Yet, women necessarily need to make a choice.

The present research aims at examining how female students choose their outfits for their graduation. In Italy, where the current research was run, thesis defense and graduation generally constitute a single, rather formal event at the end of which the student is publically declared “Laureate” (as we will explain in greater detail below). On the one hand, we investigated the effects of wearing a sexy (vs. professional) graduation outfit on observer judgments. On the other hand, we examined how students choose their graduation outfit and which image they want to convey. In so doing, we also tested the impact of objectifying TV consumption and self-objectification as potential moderators of outfit choice and of the observers’ judgments. Indeed, being exposed to objectifying media and focusing on the own appearance influence women’s self-perception and clothing choices (Ward, 2016).

### Clothing Choices and Observers’ First Impression

Clothes are elements that are used to express one’s identity (Piacentini and Mailer, 2004). Individuals have control over what they wear and are conscious of what they may communicate through clothing (Crane, 2012). Compared to men, women are believed to invest more time and money in choosing their clothes (Goldsmith et al., 2012). Moreover, women choose clothing more thoughtfully to match the social activities they have to perform (Kwon, 1997), probably because wearing an outfit that is perceived as appropriate for the occasion increases their satisfaction (Lee and Choo, 2015). Since adolescence, women decide what to wear depending on how they feel, what they want to express, and what reflects their idea of beauty (Piacentini and Mailer, 2004). Hence, clothing choices depend on how individuals perceive themselves. In fact, women’s weight, body confidence and level of self-objectification influence their clothing choice either for fashion or for comfort (Tiggemann and Andrew, 2012a,b; Grogan et al., 2013).

If clothes express an individuals’ identity, they also provide clues that observers can use to form first impressions. For instance, women wearing sensual and “revealing” clothes are usually perceived to be less competent, intelligent and moral than those women who are more fully or more appropriately dressed for the context (Gurung and Chrouser, 2007; Graff et al., 2012; Smolak et al., 2014; Daniels and Zurbriggen, 2016; Fasoli et al., 2018). In addition, women wearing provocative clothes are usually targets of negative evaluations and more likely to be blamed if they happen to be victims of sexual harassment, rape or domestic violence (Loughnan et al., 2013; Pacilli et al., 2017). On the contrary, women wearing a masculine outfit, such as a suit, are perceived to be more professional, as having better managerial skills and a higher likelihood of succeeding in a manager career (Forsythe, 1990; Thurston et al., 1990).

However, these judgments vary across contexts and situations. Glick et al. (2005) have demonstrated that women in high-status (i.e., managerial) positions are perceived to be less competent if they are dressed sexy (e.g., low-cut shirt and short skirt) rather than professional (e.g., black trousers and business jacket). In contrast, the same outfits did not have this impact on judgments of women enrolled in a low-status job (i.e., receptionists). Recently, similar effects have been observed for minimal changes in a woman’s clothes, such as skirt length and number of unfastened buttons on a blouse (Howlett et al., 2015). Hence, it seems that dressing sexy can have negative consequences on how competent women are perceived, especially when little other information is available and in professional contexts in which sexy clothing is considered inappropriate (e.g., management).

### Mass Media and Sexual Objectification

Mass media play a critical role in shaping women’s ideals and in affecting their self-perception. Over the last years, magazines have increasingly portrayed women and girls in sexualized clothes such as low cut skirts, short tops, and high-heels (Graff et al., 2013). Simultaneously, mass media – especially television – are portraying women as sexual objects by dressing them sexily (Collins, 2011). Such mass media portrayals influence women’s clothing and make-up styles (Hirschman and Thompson, 1997) and define beauty ideals that women try to reach and represent in their everyday life (Grabe et al., 2008).

Women’s portrayals in mass media are known to affect how women see themselves, their body satisfaction and self-objectification (Ward, 2016). As a consequence, Gurung and Chrouser (2007) have suggested that media exposure may also affect observers’ judgments of provocatively dressed women in everyday life. Hence, in our research we will explore whether habitual consumption of objectifying TV would affect students outfit choice and observers’ judgments of women wearing graduation outfits varying in “professionalism” vs. “sexiness”. We predict that students wearing a sexy outfit will receive more negative judgments especially from those observers who are not frequently exposed to objectifying TV. On the contrary, observers who report a high consumption of objectifying TV will not perceive sexy dressed students that negatively, because they are likely to consider women’s sexualization as normative.

At the same time, we will examine whether self-objectification plays a role on observers’ judgments of other individuals. It is well documented that mass media affect people’s self-objectification, which, in turn, impacts on people’s behaviors (Smolak and Murnen, 2011). However, to our knowledge, less is known about the extent to which individuals’ own appearance focus and body image concerns impact on how they judge other people on the basis of the clothes they wear. We expect more negative judgments of sexy dressed students by those observers who self-objectify less. In contrast, observers who self-objectify strongly should show less bias against sexy dressed students because they may perceive sexy clothing as more normative and acceptable.

### The Research Context

In Italy, graduation consists in presenting and defending a thesis in front of a committee, typically composed of 5/7 professors. This is a public and formal event attended by family and friends. The committee evaluates the student’s performance and thesis work by assigning a grade to the thesis (usually ranging from 0 to 8), which is then added to the grade point average (GPA) obtained by the students during their entire academic career. The sum of GPA and thesis points constitutes the final graduation mark that ranges from a minimum of 66 to a maximum of 110 (plus the additional honor “cum laude”). Since in such occasion the students do not wear a robe, this context provides an ideal opportunity to test how observers evaluate a female student and her work depending on the outfit she chooses for a formal event like her graduation. At the same time, it also offers an opportunity to examine how female students choose to dress depending on the impression they want to make and on their personal characteristics. Moreover, Italy, compared to other European countries, has a significant history of objectifying mass media (CENSIS, 2006; see also Volpato, 2011) both in Italian advertisements and TV (Guastini et al., 2014). Hence, Italy represents an ideal context to investigate whether chronic exposure to objectifying TV has an impact on TV-consumers’ judgments and choices.

### Overview of Research

Across three studies, we examined how women choose their graduation outfit and what inferences observers draw on the basis of their outfit. In so doing, we extend previous research that merely examined work-related situations (Glick et al., 2005; Howlett et al., 2015) to a context (i.e., university) where students’ outfits are often question of debate and where competence and diligence are highly valued. In Study 1, we investigated whether the graduation outfit worn by female students would impact the observers’ perception of the students’ competence and sexiness, two aspects that are typically tested in research on women’s sexualization. Also, we examined whether and how the outfit influenced the perception of students’ effort and final outcomes. Hence, we not only focused on person perception, but we move forward to test how the attire influenced expectations about the students’ diligence and ability of graduating successfully.

Moreover, we considered the observer’s perception of different observer samples varying in age, status, and experience with Thesis committees. Students’ attires may indeed be perceived very differently by people who have different fashion styles, belong to different generations, have different backgrounds and experiences (young female peers and adults), or hold different roles (students and professors). Students and professors are known to have different opinions about clothing, with faculty members favoring students’ conservative clothing and students preferring trendy clothing (Ruetzler et al., 2012). Also, a recent study (Cabras et al., 2018) has shown that teachers perceive sexualized (vs. non-sexualized) students as more likely to engage in behavior impeding learning (e.g., not paying attention in class, copying homework, etc.). Thus, the inclusion of female peers, adults from the general population, and professors allowed us to gauge the generality or specificity of reactions to professional vs. sexy clothing.

In Study 2, we examined the reasons behind students’ outfit choices and, importantly, we tested observers’ impressions with reference to students’ outfit used in real life. In particular, we asked students who had recently graduated to indicate what motivated them to choose their graduation outfit. At the same time, the students and their outfits were evaluated by a group of observers who also guessed the final mark obtained by the students. This allowed us to test the link between students’ outfit and observers’ perceptions. In Study 3, we extended results of Study 1 by using photos of real female graduates wearing professional or sexy outfits, providing generalizability and higher ecological validity to our research. Finally, across studies, we explored the role of objectifying TV consumption and self-objectification in guiding the observers’ judgments and the students’ outfit choices.

## Study 1

In Study 1 we tested whether students’ outfit would influence observers’ judgments. We predicted that students wearing a professional outfit would be judged as more competent (and as considering competence more important; Hypothesis 1) and as less sexy (and as considering appearance less important; Hypothesis 2) than those wearing a sexy outfit. Moreover, we expected that, the professionally (vs. sexily) dressed students would be thought to have earned better grades for their thesis, resulting in a higher overall graduation mark (Hypothesis 3). We also hypothesized that observers would expect professionally dressed students to have produced a thesis of higher quality (Hypothesis 4) and to be more likely to succeed in their future careers (Hypothesis 5). Importantly, these hypotheses were tested on three different samples, namely female student peers, an adult population, and university professors. Hence, we explored differences due to age and status on observers’ judgments.

### Methods

#### Participants

Six hundred and sixty-seven participants accessed the survey. Inclusion criteria to define the final sample were the following: (a) being Italian, (b) not knowing the person in the picture, and (c) having completed the survey in all of its parts. The final sample consisted of 573 participants. In particular, the sample involved 223 female university students (“female peer” sample, *M_age_* = 20.66, *SD* = 1.81), 295 adults (“adult” sample, 104 men, 3 unknown; *M*_age_ = 48.58, *SD* = 8.56) and 55 university professors (“professor” sample, 25 men, *M*_age_ = 51.11, *SD* = 10.11). The majority of the three samples came from Northern Italy (73.5%, *n* = 421), was Catholic (57.1%, *n* = 327), not politically identified (34.9%, *n* = 200) or left/center-left (27.1%, *n* = 155). Students were all undergraduate and mostly unemployed (83.6%, *n* = 179), all professors held a Ph.D., and the majority of the adult sample had a high school diploma or higher education (76.5%, *n* = 178) and was employed (71.9%, *n* = 212).

The majority of professors (81.5%) indicated that, in their Schools, the student’s evaluation and graduation takes places immediately after the defense. Moreover, they neither strongly agreed nor strongly disagreed with the fact that students of their Schools usually wear professional graduation outfit [*M* = 3.54, *SD* = 1.44; *t*-test against midpoint (4) on a scale from 1 = completely disagree to 7 = completely agree: *t*(53) = −2.37, *p* = 0.02] and they did not find, on average, students’ outfits either very appropriate or inappropriate to the context [*M* = 4.04, *SD* = 1.54; *t*-test against midpoint: *t*(53) = 0.17, *p* = 0.86].

#### Procedure

Participants were recruited through students’ contacts and social networks, with the exception of the professors who were recruited among contacts of the researchers. Participants were invited to take part in a study about first impressions and provided with a link to an online survey. After consenting to participate in the study, they reported their demographic information (i.e., gender, age, nationality, geographical background, occupational status, religion and political orientation), were introduced to the study, and then viewed a photo of a female student in her graduation outfit. The cover story mentioned that the student obtained a M.Sc. degree and the picture portrayed her right before the defense. Participants were randomly exposed either to a student in a sexy or in a professional outfit (with pictures randomly taken from the pool of materials described below). Then, they were asked to rate the student’s performance and career success. Next, they rated the student and indicated their outfit choice. Finally, they completed the Objectified Body Consciousness Scale and objectifying TV consumption scale. At the very end, before being debriefed and thanked, participants indicated whether they knew the person portrayed in the picture (yes/no) and the degree of certainty (from 1 = *not at all* to 7 = *completely*).

#### Materials

##### Photos

Photos of 37 female students (*M*_age_ = 22.59, *SD* = 1.49) were taken to assure a broad range of stimuli. Photographed students were all White, all Italian except for one, and mostly coming from Northern Italy (77.8% of participants). Following previous research on this topic (Glick et al., 2005; see also Lennon et al., 2017), we considered two types of outfit: “sexy” outfits were provocative and revealing clothes and “professional” outfits consisted of business and formal clothing. Thus, each student volunteer was asked to dress up with two different outfits, namely a *professional* (e.g., trousers or semi-long skirt, shirt covering neckline and breast, jacket, and flat shoes) and a *sexy* outfit (e.g., short skirt or mini dress, evident neckline, and high heels). Pictures of the person wearing the two outfits were taken in the same position (i.e., frontal, standing and with a thesis in her hands) and from the same distance. Faces were blurred in order to protect the person’s privacy and to avoid effects of other appearance related features (e.g., make-up). The background was always a white wall. For the female peer and adult samples, all 74 photos were used, whereas for the professor sample we selected 4 target students shown either in a professional and sexy outfit, resulting in eight photos to which participants were randomly assigned. Photographed students also judged their own outfits as reported in the Supplementary Information S1.

##### Students’ perception

Participants rated the student’s competence (competent, intelligent; α = 0.81), sexiness (sexy, attractive; α = 0.83), and beauty (elegant, fashionable, and feminine; α = 0.67). They then indicated how much they believed the student cared about appearing competent and intelligent (importance of competence, α = 0.85), and about appearing beautiful, elegant, feminine, fashionable (importance of beauty; α = 0.89). Answers were provided on a scale from 1 (*not at all*) to 7 (*completely*). Indexes were created by averaging the ratings referring to each variable.

##### Students’ performance

Participants were asked to guess the number of points (from 0 to 8) the student had earned for her thesis and her final mark (from 66 to 110, where “66” represents the minimum a student could obtain as a final mark in order to graduate). Participants also made guesses about the student’s thesis work by answering three questions (i.e., “how much effort do you think she put in her thesis work?”, “How logical and organized do you think her thesis is?”, “How original do you think her thesis is?”). Answers were provided on a scale from 1 (*not at all*) to 7 (*completely*). An index of thesis evaluation (α = 0.87) was calculated by averaging the ratings for the three items. The higher the score, the better the performance.

##### Career success

Participants indicated the likelihood that the student would have a successful career by responding to two items (i.e., “How easily do you think she will find a job?”, “How successful do you think she will be in her career?”). Answers were provided on a scale from 1 (*not at all*) to 7 (*completely*). An index of career success (α = 0.82) was calculated by averaging the ratings for the two items with higher scores indicating a higher likelihood of success.

##### Outfit choice

Participants also indicated the likelihood that they would have worn that outfit if they were the student on a scale from 1 (*not at all*) to 7 (*completely*).

##### Objectified body consciousness scale

Participants – with the exception of the professors – completed the 18 items of the body shame and body surveillance subscales of the Objectified Body Consciousness Scale (OBCS; McKinley and Hyde, 1996). Answers were provided on a scale from 1 (*not at all*) to 7 (*completely*). Since the same pattern of results emerged when considering the two subscales, an index of self-objectification (α = 0.87) was calculated, with higher scores indicating higher self-objectification.

##### Objectifying TV consumption

Participants indicated how frequently they watched popular TV shows on a scale from 1 (*never*) to 4 (*always*), with an additional response option indicating that they did not know the program. The TV shows had been pretested (*n* = 38 men and *n* = 35 women) by asking participants to judge the degree to which the show was objectifying (i.e., portraying women as sexual objects or as ornaments) and paying attention primarily to women’s bodies rather than to their personality (responses’ scale from 1 = *never* to 4 = *always*). Eight of the TV shows were classified as objectifying (*M* = 3.17, *SD* = 0.67) and 8 as non-objectifying (*M* = 1.50, *SD* = 0.79). Five additional TV shows, located in the middle range (*M* = 2.93, *SD* = 0.79), were included as fillers but not considered in the analyses. The three groups of TV shows were all significantly different from each other, *t*s > 7.19, *p*s < 0.001. An objectifying TV consumption index was calculated by subtracting the frequency of watching non-objectifying TV from that of objectifying TV. Thus, the higher the score, the higher was the consumption of objectifying TV.

### Results

#### Effects of Clothing on Observer Ratings

ANOVAs were run for each dependent variable, including outfit (professional vs. sexy), participants’ gender (male vs. female) and sample (female peer vs. adults vs. professors) as between-participants variables. Effects, means and standard deviations by experimental condition on each dependent variable are reported in Table 1.

**Table 1:** Cronbach’s alpha, means and standard deviation, and main effects of outfit on all the dependent variables (Study 1).

	Sexy *M* (*SD*)	Professional *M* (*SD*)	*F*-values
Thesis points	5.80 (1.38)	6.29 (1.14)	*F*(1,540) = 16.59, *p* < 0.001, 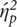 = 0.030
Final mark	96.07 (11.53)	99.27 (8.86)	*F*(1,550) = 6.52, *p* = 0.011, 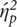 = 0.028
Thesis evaluation	4.60 (1.25)	5.22 (1.06)	*F*(1,558) = 12.04, *p* = 0.001, 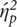 = 0.021
Career success	4.26 (1.20)	4.72 (1.16)	*F*(1,551) = 2.74, *p* = 0.098, 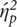 = 0.005
Competence	4.43 (1.24)	4.95 (1.12)	*F*(1,556) = 10.34, *p* = 0.001, 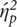 = 0.018
Sexiness	4.53 (1.59)	3.79 (1.39)	*F*(1,558) = 12.21, *p* = 0.001, 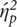 = 0.021
Beauty	3.73 (1.54)	3.82 (1.41)	*F*(1,559) = 0.67, *p* = 0.41, 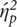 = 0.001
Importance of competence	4.62 (1.62)	5.58 (1.23)	*F*(1,557) = 25.87, *p* < 0.001, 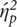 = 0.044
Importance of beauty	5.38 (1.11)	4.31 (1.28)	*F*(1,557) = 36.85, *p* < 0.001, 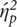 = 0.062
Outfit choice	1.70 (1.20)	2.78 (1.80)	*F*(1,555) = 24.47, *p* < 0.001, 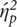 = 0.042

*Different degrees of freedom are due to missing values.*

Significant effects of outfit were found on all dependent variables with the exception of beauty and career success. In particular, confirming hypotheses 1 and 2, participants judged the target students in a professional outfit as being more competent and less sexy, and as giving more importance to competence and less to beauty than when the outfit was sexy. Moreover, confirming hypothesis 3, participants attributed more thesis points and a higher final mark to the students when wearing a professional rather than a sexy outfit. The same students were rated as having put more effort into their thesis work when dressed professionally, as predicted by hypothesis 4. Finally, participants also indicated that they would have chosen the professional rather than the sexy outfit if they were the student.

Interestingly, a significant interaction between outfit and sample emerged, *F*(1,551) = 3.11, *p* = 0.045, 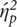 = 0.01, for career success. Pairwise comparisons (Bonferroni’s correction) indicated that female peers perceived the professionally dressed students (*M* = 4.73, *SD* = 1.16) as more likely to find a job and to have a successful career than when the same students were presented with a sexy outfit (*M* = 4.26, *SD* = 1.20; *p* = 0.008). On the contrary, adults (*M*_professional_ = 4.03, *SD* = 1.38 vs. *M*_sexy_ = 4.33, *SD* = 1.37; *p* = 0.03) judged the students wearing a sexy outfit as having higher chances of success. Professors (*M*_professional_ = 4.23, *SD* = 1.20 vs. *M*_sexy_ = 4.87, *SD* = 1.00; *p* = 0.09) showed a non-significant trend in the same direction.

No significant effects or interaction with gender were found (*F*s < 3.28, *p*s > 0.07). The only gender difference was on outfit choice with men (*M* = 3.07, *SD* = 1.92) reporting they would have chosen the sexy outfit slightly more than women (*M* = 2.36, *SD* = 1.81) if they were the student, *F*(1,555) = 3.81, *p* = 0.05, 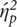 = 0.01.

#### Mediation Analyses

Previous studies (Glick et al., 2005; Howlett et al., 2015) merely tested whether clothing affected the target’s perceived competence. Here, we wanted to explore whether such perception could affect perception of the student’s performance. Hence, two mediation analyses predicting an impact of outfit on final mark and thesis points via perceived competence were run by using Hayes’ (2013) PROCESS macro (Model 4) for SPSS and bias-corrected intervals (5000 bootstrap resamples). The analysis on thesis points showed that professional outfit was associated with higher perceived competence (*b* = 0.25, *SE* = 0.05, *t* = 4.92, *p* < 0.001) and competence was also positively associated with thesis points (*b* = 0.42, *SE* = 0.04, *t* = 10.13, *p* < 0.001). Supporting a partial mediation, the direct effect of outfit on thesis points (*b* = 0.24, *SE* = 0.05, *t* = 4.46, *p* < 0.001) was reduced when considering competence as mediator (*b* = 0.13, *SE* = 0.05, *t* = 2.67, *p* = 0.008). Confirming the mediated path, the indirect effect was statistically significant (*a^∗^b* = 0.10, *SE* = 0.02, 95% CI [0.06, 12)] as was the Sobel test (*z* = 4.41, *p* < 0.001). Similarly, the direct effect of outfit on final mark (*b* = 1.57, *SE* = 0.44, *t* = 3.60, *p* = 0.003) was reduced when entering competence as mediator (*b* = 0.93, *SE* = 0.43, *t* = 2.17, *p* = 0.030). The significant indirect pathway of students’ outfit on final mark was mediated by perceived competence (*a^∗^b* = 0.65, *SE* = 0.15; 95% CI [0.38, 0.99]; *z* = 4.12, *p* < 0.001). Together, these results indicate that a sexy attire decreased perceived competence which, in turn, led observers to imagine that the student must have performed worse.

#### The Role of Exposure to Objectifying Television on Observer Ratings

Overall participants reported low levels of objectifying TV consumption and a preference for non-objectifying over objectifying TV (*M* = −0.27, *SD* = 0.41). A univariate ANOVA showed that this was especially true for adults (*M* = −0.34, *SD* = 0.39) and professors (*M* = −0.39, *SD* = 0.25) and less so for young female students (*M* = −0.14, *SD* = 0.43), *F*(1,560) = 15.09, *p* < 0.001, 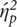 = 0.051. This effect may be explained by age; in fact when entering this variable as a covariate in the analysis, age was the only significant predictor of objectifying TV consumption, *F*(1,558) = 4.08, *p* = 0.044, 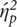 = 0.007.

Next, we ran a moderation analysis on each dependent variable to test whether participants’ consumption of objectifying TV moderated the effects of outfit (−1 = sexy and 1 = professional) on the perception of students and of their graduation outcomes. Analyses were performed using Hayes’ (2013) PROCESS macro (Model 1) and bias-corrected intervals (5000 bootstrap resamples), introducing the mean centered moderator and controlling for the participants’ gender and age as covariate. Among all analyses only one significant result involving objectifying TV consumption was found. Specifically, the interaction term between outfit and objectifying TV consumption was significant for thesis points (*b* = −0.13, *SE* = 0.05, *t* = −0.2.41, *p* = 0.01). Participants low in objectifying TV consumption estimated more thesis points obtained by the professionally than by the sexy dressed students (*b* = 0.37, *SE* = 0.08, *t* = 4.86, *p* < 0.001), whereas participants high in objectifying TV consumption did not differentiate between thesis points attributed to professionally and sexy dressed students (*b* = 0.11, *SE* = 0.08, *t* = 1.46, *p* = 0.14).

#### The Role of Self-Objectification on Observer Ratings

Participants reported overall moderately low levels of self-objectification (*M* = 3.54, *SD* = 0.93), with the female peer sample (*M* = 3.80, *SD* = 0.87) reporting greater self-objectification than adult participants (*M* = 3.28, *SD* = 0.90), *F*(1,446) = 8.85, *p* = 0.003, 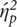 = 0.019. The same analyses as above were conducted using self-objectification (mean centered) instead of objectifying TV exposure as moderator variable, while controlling for gender and age. We will only report significant findings involving a self-objectification moderation effect.

A significant interaction was found for thesis points (*b* = −0.12, *SE* = 0.06, *t* = −2.06, *p* = 0.04), suggesting that low self-objectified participants attributed more thesis points to the professionally than to the sexy dressed students (*b* = 0.33, *SE* = 0.08, *t* = 3.94, *p* < 0.001), whereas no difference was found for participants with higher level of self-objectification (*b* = 0.08, *SE* = 0.08, *t* = 1.03, *p* = 0.30). Similarly, as shown by the significant interaction on students’ career success (*b* = −0.14, *SE* = 0.06, *t* = −0.2.29, *p* = 0.03), low self-objectified participants perceived the students wearing a professional outfit as more likely to be successful in their career than when the same students were dressed in a sexy fashion (*b* = 0.22, *SE* = 0.09, *t* = 2.51, *p* = 0.01). On the contrary, high self-objectified participants did not perceive differences in success likelihood depending on how the students were dressed (*b* = −0.06, *SE* = 0.09, *t* = −0.73, *p* = 0.47).

Interestingly, the interaction effect on perceived competence (*b* = −0.11, *SE* = 0.06, *t* = −2.04, *p* = 0.04), showed that all participants perceived the professionally dressed students as more competent than the sexy ones, but the effect was more evident for those reporting low (*b* = 0.40, *SE* = 0.08, *t* = 5.09, *p* < 0.001) than high levels of self-objectification (*b* = 0.17, *SE* = 0.08, *t* = 2.20, *p* = 0.028). The significant interaction on perceived sexiness (*b* = −0.16, *SE* = 0.07, *t* = −2.22, *p* = 0.026) showed the opposite pattern: high self-objectified participants (*b* = −0.53, *SE* = 0.10, *t* = −5.14, *p* < 0.001) judged the students sexier when dressed in a sexy rather than professional way than participants low in self-objectification did (*b* = −0.20, *SE* = 0.10, *t* = −1.98, *p* = 0.047).

### Discussion

Study 1 illustrates the risks associated with sexy clothing during thesis defense and graduation. Women were judged approximately equally beautiful regardless of outfit, which may not be surprising given that they were the same women photographed in identical poses. Provocatively dressed women were generally judged as more sexy and as caring more about their appearance. However, when students dressed sexy, they were generally thought to perform worse (they were associated with less thesis points, a lower final mark, and a thesis of lower quality) and were perceived as less competent and as caring less about competence. Importantly, these effects emerged for all participant groups, indicating that female peers, adults and professors made similar judgments based on the attire. Hence, no group seems to be immune to the outfit bias. Importantly, our mediational analyses suggest that participants “penalized” students dressed sexy because they perceived them as less competent (see Cabras et al., 2018).

The only variable judged differently by the three groups was anticipated career success. Whereas female peers thought that the professional attire would facilitate finding a job and succeeding in a career, the general adult population and the professors (albeit the trend was non-significant in the latter case) thought that the sexy attire was advantageous. This result is open to different interpretations: students may believe that dressing professionally not only conveys the idea of being competent and skilled, but also communicates the ability to conform to job norms. Hence, for young people, communicating competence (rather than sexiness) through clothing should be advantageous. Adults, instead, may believe that looking sexy implies advantages when applying for a job and this is why they associated a sexy outfit with higher career success likelihood. However, these are only speculations and whether the observed differences reflect generational or age differences remains an open question for future research.

Study 1 also investigated two potential moderators of the outfit effects, namely exposure to objectifying TV and the degree of self-objectification. TV consumption turned out to be relevant only for thesis points’ attribution. Only participants with low levels of objectifying TV consumption thought that the sexy (vs. professionally) dressed student had obtained fewer thesis points. The same pattern was found for participants low in self-objectification. Furthermore, self-objectification exerted effects on other dependent variables. The less participants self-objectified, the more they rated the professionally dressed students as competent and the more optimistic they were about their career perspective. Together, the moderating function of TV consumption and self-objectification was limited to a few dependent variables, but, where moderation was found, a consistent pattern emerged. In all cases, participants less exposed to objectifying TV and with lower levels of self-objectification attributed greater competence and success to women dressed in a professional rather than sexy fashion, thus showing a stronger outfit bias. Participants with high objectifying TV consumption and high self-objectification were less likely to distinguish between sexy and professional attire, possibly because they consider sexy clothing as more “normative.”

## Study 2

Study 2 moved to the “real world” and investigated how students choose their graduation outfits and how these clothes are perceived by observers. In particular, we asked female students who had graduated in the last few years to provide a photo portraying them on the graduation day along with ratings concerning their outfit. Different participants were then exposed to these pictures and asked to draw inferences about the students and to evaluate their attire.

Hence, we extended our previous findings in different ways. First, we explored how graduation outfits are chosen in real life and which student characteristics relate to this choice. In particular, we predicted that students with lower self-objectification and lower consumption of objectifying TV, and those who attribute greater importance to competence will be more likely to have chosen an outfit that was expressing their competence rather than their beauty or personality (Hypothesis 1). Second, we examined what inferences external observers would draw about these students and whether impressions about the students and their outfits would affect observers’ expectations of obtained thesis points and final mark. In line with Study 1, we expected that a perception of the student as more competent and less sexy would predict an estimation of more thesis points and a higher final mark (Hypothesis 2a). Moreover, a perception of their outfit as more appropriate was predicted to be linked to higher estimated final mark and thesis points (Hypothesis 2b).

Third, we tested whether the perception of the students’ outfit could explain their actual thesis points that determined the students’ final mark. We were interested in examining whether the outfit perception would predict the evaluation given by the committee. If the outfit affected the raters’ estimation of thesis points (as already shown in Study 1), then the committee may have been influenced in much the same way, assigning more thesis points to the professionally dressed students. In particular, we not only asked our raters to judge how appropriate the outfit was for the occasion, but also how sexy and masculine it was. We relied on (a) appropriateness as it was an important aspect reported by students themselves (see Supplementary Information S2), (b) on sexiness because findings of Study 1 showed a negative effect of sexy outfits on observers’ judgments, and (c) on masculinity as research has shown that a more masculine outfit (e.g., suit) is preferred in more formal contexts and conveys greater competence (Kelan, 2013). Hence, we predicted that perception of the outfit as more appropriate, masculine and less sexy would be positively associated with more thesis points obtained by the student (Hypothesis 3). This prediction should not regard the pre-defense GPA based on exams since it should not be affected by graduation outfit.

### Methods

#### Participants

##### Phase 1

In this phase, 114 out of 140 female students (*M*_age_ = 25.32, *SD* = 2.32) completed a survey, provided their graduation photos and consented to their use in our research. The majority of them (68.4%, *n* = 78) graduated in Humanities (e.g., foreign languages) and received a Master’s degree (64%, *n* = 73). Also, they were mostly from Northern Italy (77.2%, *n* = 88), Catholic (66.7%, *n* = 76), and of varied political orientation (41.6% of participants, *n* = 47, preferred not to answer this question). Moreover, 26.5% of participants, *n* = 30, were currently continuing their studies, whereas half of the remaining sample was employed (50.9%, *n* = 58).

##### Phase 2

In the second phase of the study, after excluding 5 non-Italian participants and 8 participants who indicated they knew one or more persons portrayed in the pictures, we had a sample of 383 Italian participants (177 men, *M*_age_ = 27.73, *SD* = 9.01) rating the students in the photos. The sample was mostly from Northern Italy (78.0%, *n* = 296) and Catholic (57.9%, *n* = 220), with approximately half of the participants having a left or center-left political orientation (49.3%, *n* = 185) and being employed (45.8%, *n* = 172).

##### Phase 3

In the third phase of the study, four psychology interns (three female and one male student, *M*_age_ = 22.25, *SD* = 0.96) rated the outfit of students portrayed in all photos of Phase 1.

#### Materials and Procedure

##### Phase 1

Participants were recruited through students’ contacts and, if necessary criteria (i.e., recent graduation, defense in front of a committee, pictures with a visible outfit) were fulfilled, they provided a photo of themselves during their graduation defense. Then, they completed the following measures and reported their demographic information (i.e., age, nationality, geographical background, religion, political orientation and employment status) in an online survey.

###### Performance

Participants reported their GPA that testified their academic career before the defense as well as the thesis points and the final mark obtained after the defense.

###### Outfit perception

Participants indicated to what degree they thought the chosen attire represented their *competence* (e.g., competence, being professional; α = 0.72), their *beauty* (i.e., beauty, body shape, femininity, elegance, and fashion; α = 0.78), and their *personality* (i.e., expressing their personality, making them feel at ease, α = 0.83). All answers were provided on a scale from 1 (*not at all*) to 7 (*completely*). Indexes of outfit perception were calculated by averaging the ratings for each dimension. Participants were also asked to indicate whether they had chosen the attire autonomously, which was true in 80% of the cases and to answer an open-ended question specifying how and why they had chosen their outfit (see Supplementary Information S2).

###### Self-perception

Participants reported how much appearing *competent* (i.e., intelligent, competent; α = 0.58) or *beautiful* (i.e., beautiful, elegant, fashionable, elegant, feminine; α = 0.76) was important for them. All answers were provided on a scale from 1 (*not at all*) to 7 (*completely*). Indexes of outfit perception were calculated by averaging the ratings for each dimension.

###### OBCS and objectifying TV consumption

Participants completed the OBCS (α = 0.83) and objectifying TV consumption scale (α = 0.83). Indexes were calculated as in Study 1.

##### Phase 2

Participants were recruited through students’ contacts. Each participant was exposed to 6 pictures randomly selected from the pool of photos provided by students in Phase 1. As in Study 1, for each picture, participants filled in the measures below, reported the same demographic information as in Phase 1 and indicated whether they knew any of the persons portrayed in the photos (yes/no).

###### Student performance

Participants guessed the number of thesis points (from 0 to 8 where the minimum corresponded to a poor and the maximum to an excellent evaluation) and the final mark (from 66 to 110) obtained by the student after the defense.

###### Student perception

Participants rated the students’ competence (“how competent/intelligent is the student?”) and sexiness (“How sexy/attractive is the student?”) on a scale from 1 (*not at all*) to 7 (*completely*). Two indexes were calculated by averaging the ratings referring to each variable.

###### Outfit appropriateness

A single-item (“How appropriate is the outfit for the thesis defense?”) was used. Answers were provided on a scale from 1 (*not at all*) to 7 (*completely*).

##### Phase 3

The raters, blind to the hypotheses of the research, judged the attire of all 114 photos on masculinity, appropriateness, and sexiness. The advantage of this procedure was that the raters saw the entire range of photos rather than only a subset and judged the outfit instead of the student. According to Landis and Koch (1977; see also Cicchetti and Sparrow, 1981), all raters showed a good internal agreement (ICC) on outfit masculinity ratings (ICC = 0.63), but only a fair (ICC = 0.42) and a poor (ICC = 0.34) agreement on outfit appropriateness and sexiness, respectively. Thus, for the last two dimensions, we decided to consider only ratings of two raters (a male and a female) that showed an adequate agreement as it is important to have ratings that refer to the same idea of outfit sexiness and appropriateness. Note, however, that the same pattern of results emerged when using average scores of all four raters.

###### Outfit masculinity

A single item measured the perceived masculinity associated with the outfit (i.e., “How feminine vs. masculine do you consider this outfit?”). Answers were provided on a scale from 1 (*very feminine*) to 7 (*very masculine*). Inter-rater agreement between the four raters was good (ICC = 0.63).

###### Outfit appropriateness

As in Phase 2, a single item (i.e., “How appropriate do you consider this outfit for a thesis defense?”) from 1 (*not at all*) to 7 (*completely*) was used. Inter-rates agreement among two raters was good (ICC = 0.61).

###### Outfit sexiness

A single item measured perceived sexiness of the outfit [i.e., “How professional vs. sexy do you consider this outfit?” from 1 (*very professional*) to 7 (*very sexy*)]. Inter-rates agreement was good for two raters (ICC = 0.70).

### Results

Analyses were performed in three steps, according to the study phases and hypotheses.

#### Phase 1: Self-Perception

First, we focused on female students’ perceptions of themselves and their outfit as revealing their competence, personality, or beauty. A repeated measures ANOVA, *F*(2,226) = 12.48, *p* < 0.001, 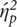 = 0.10, showed that, on average, participants chose their outfits more to express their personality (*M* = 5.07, *SD* = 1.53) and to appear beautiful (*M* = 4.93, *SD* = 4.55) than to appear competent (*M* = 4.55, *SD* = 1.41; *p*s < 0.002). To understand what drove their motivation to choose an outfit expressing one or the other dimension, we tested whether self-objectification (OBCS), objectifying TV consumption and importance of competence over beauty (calculated by subtracting the subjective importance of appearing beautiful from that of appearing competent) predicted perceptions of the chosen outfit as (a) expressing the student’s competence (over beauty), and (b) her personality and feeling at ease. All scores were z-transformed. The only model that turned out to be significant regarded the perception of the outfit as expressing the student’s personality. The model [*R*^2^ = 0.08, *F*(3,96) = 2.70, *p* = 0.05], showed that both self-objectification (β = −0.22, *t* = −2.05, *p* = 0.04) and importance of competence over beauty (β = −0.29, *t* = −2.66, *p* = 0.009) negatively predicted the students’ outfit ratings as expressing their personality. This suggests that participants low on self-objectification and those who cared less about appearing competent chose an outfit that fit their personality and made them feel at ease, in partial support of Hypothesis 1.

#### Phase 2: Observers

As a second step, we tested whether observers’ impressions of the students as competent and sexy and of their outfits as appropriate for the occasion affected their expectations about the estimated thesis points and final mark obtained. Hence, we used observers’ ratings obtained in Phase 2, and ran analyses considering the 114 students/photos as unit of analysis.

Regression analysis on estimated thesis points [*R*^2^ = 0.60, *F*(3,110) = 55.40, *p* < 0.001] yielded a significant effect of outfit appropriateness (β = 0.76, *t* = 11.64, *p* < 0.001), indicating that the more appropriate the outfit was considered for the occasion, the higher the number of thesis points attributed to the students by observers. The same analysis performed on the estimated final mark [*R*^2^ = 0.51, *F*(3,110) = 38.74, *p* < 0.001] showed a significant negative effect of sexiness (β = −0.20, *t* = −2.81, *p* = 0.006) suggesting that the more sexy the students were judged, the lower the estimated final mark. Moreover, a significant effect of outfit appropriateness (β = 0.77, *t* = 10.64, *p* < 0.001) indicated that the more appropriate the attire was judged for the occasion, the higher the estimated final mark. Overall, although no significant effects of perceived competence were found, these findings supported Hypotheses 2a and 2b indicating that observers expected more sexy students to have obtained lower final marks, but it also showed that appropriateness of outfit specifically mattered to them when making inferences.

#### Phase 3: Raters

Overall, the more sexy the students’ attire was judged, the less appropriate for the occasion, *r*(114) = −0.493, *p* < 0.001, and the less masculine it was perceived, *r*(114) = −0.491, *p* < 0.001; in contrast masculinity and appropriateness were unrelated, *r*(114) = 0.027, *p* = 0.78.

We then tested whether students with less sexy, more masculine or more appropriate outfits (as judged by the raters) had actually been assigned more thesis points by their respective committees. Due to the great variance in the points typically assigned to a thesis both between subject areas and between B.Sc. and M.Sc. level, we limited this part of the analyses to the five subject areas with comparable standards (i.e., social and political sciences, communication, education, psychology and humanities), while excluding those areas that had significantly higher or lower mean Thesis points (*p* < 0.05). Also, we excluded students from this analyses if their GPA exceeded 105 points, given that the number of points that can be assigned to students with such excellent GPAs are by definition limited (for instance a student with a GPA of 108 can at most receive an additional 2 thesis points, given that the maximum grade is 110). For the 5 comparable subject areas we then standardized both the GPA and the Thesis points separately for BS and for MS thesis. In line with Hypothesis 3, fewer thesis points were assigned to students whose attire was judged as more sexy (β = −0.64, *t* = −2.55, *p* = 0.019) and, as a non-significant tendency, as more masculine (β = −0.36, *t* = −1.89, *p* = 0.066), after controlling for GPA [β = 0.27, *t* = 1.81, *p* = 0.079; Model: *R*^2^ = 0.22, *F*(3,38) = 2.73, *p* = 0.043]. As expected, GPA was not predicted by rated appropriateness, masculinity or sexiness of the outfit worn on the day of the defense (all *p*s > 0.86).

To investigate whether the rater judgments reflected the self-presentation intentions of the students, we ran three regression analyses using, one at a time, the ratings of appropriateness, masculinity and sexiness as dependent variable and the students’ self-reported importance of competence, perception of outfit as expressing competence and personality as predictors (all transformed into *z*-scores). The only significant model [*R*^2^ = 0.10, *F*(3,94) = 3.66, *p* = 0.01] referred to sexiness of the outfit. The less the students intended to communicate competence, the more the attire was rated as sexy (β = −0.40, *t* = −3.08, *p* = 0.003).

### Discussion

Study 2 extended previous findings by showing that female students chose their graduation outfits mostly to appear beautiful and to express their personality, followed by a lower motivation to appear competent. Moreover, choices of graduation outfits were influenced by students’ level of self-objectification but not by their consumption of objectifying TV. Indeed, we only found that students who rarely self-objectified, and those who cared less about appearing competent rather than beautiful, had chosen outfits so as to express their personality and to feel at ease.

Regardless of how the students chose their outfit, Study 2 demonstrated once again that graduation outfit affected the observers’ judgments. Extending results of Study 1, we found that students wearing clothing rated as appropriate for the occasion were also perceived as having performed better and hence having obtained more thesis points and better final marks, whereas students perceived as sexier were associated with lower final marks. This latter result concerned judgments on the students’ sexiness (rather than on their outfit) and seems to suggest that observers believed that a sexier student may have been penalized because of her appearance.

To test if this was the case in real life, we examined whether outfit appropriateness, masculinity and sexiness affected actual obtained thesis point. We found that the attire, as judged by our independent raters, was predictive of the actual number of thesis points earned by the student (even after controlling for GPA). In particular, students being judged as wearing a sexier (and, as a non-significant tendency, also a more masculine outfit) obtained fewer thesis points. Thus, although masculinity and sexiness are negatively correlated, both too sexy and too masculine looks seem to create disadvantages. Although analyses on thesis points are limited to a subsample, our findings suggest that the outfit worn by the student may influence the committee’s attribution of thesis points. Obviously the current study, differently from Study 1, does not speak to causality, so we cannot conclude with certainty that students were penalized by the committee for dressing too sexy. However, this remains a possible explanation.

Together, the findings of this study indicated that students’ outfit choices are related to how they are perceived and how their performance is evaluated. In particular, this study showed that outfit appropriateness for the occasion is an important dimension. However, what seems to be most relevant in reality is how sexy and, to a lesser degree, how masculine the outfit is perceived. Whereas observers believed that, with a more appropriate outfit, the student would have earned more thesis points, in reality it is the less sexy (but not too masculine) outfit that leads to more thesis points.

## Study 3

In Study 3 we extended previous results in different ways. First, we wanted to replicate the findings of Study 1 using real pictures to assure that the effects observed in Study 1 were not an artifact of a highly polarized clothing manipulation. Moreover, Study 2 has shown that the perceived outfit sexiness and masculinity can both predict actual thesis point attribution. Hence, Study 3 experimentally tested whether real outfits that were perceived as sexy (vs. professional) and as masculine (vs. feminine) would affect the perception of students, of their outfits, and of their performance.

We selected a subsample of the photos of Study 2 as stimuli, namely photos of students wearing a professional and masculine outfit (e.g., jacket and trousers) and of students with a sexy outfit (e.g., mini-dress, short skirt) creating an experimental situation similar to Study 1. Hence, we tested observers’ perceptions of the students’ competence and sexiness, and estimated final mark and thesis points. This allowed us to test the casual link between outfit and observers’ perception. We predicted that, compared to students in sexy clothing, students wearing professional outfits would be judged as having obtained higher final marks and more thesis points (Hypothesis 1) and as being more competent and less sexy (Hypothesis 2). We also predicted that a student in a professional outfit would be perceived as more appropriately dressed than those in a sexy outfit (Hypothesis 3). Moreover, we extended previous findings by testing whether appropriateness of the outfit affected the perceived student’s competence, which, in turn, explained performance expectations in terms of thesis points and final mark. Finally, as in Study 1, we tested the impact of observers’ self-objectification and objectifying TV consumption.

### Methods

#### Participants

Seventy-three Italian participants (39 men, *M*_age_ = 27.58, *SD* = 9.50) completed the online survey in all of its parts and did not know any of the persons portrayed in the pictures. They were mostly coming from Northern Italy (78.1%, *n* = 57), half of them were Catholic (52.1%, *n* = 38), unemployed (53.2%, *n* = 39), and with a left or center-left political affiliation (48.6%, *n* = 35).

#### Procedure

Participants were recruited via email or social networks and exposed to the same cover story and information of Study 1. Before being exposed to the pictures, participants reported their demographic information including gender, age, nationality, geographical background, religion, political orientation and employment status. Next, they were randomly exposed to 6 pictures portraying M.Sc. female students during their graduation day, 3 of whom were wearing a professional and 3 a sexy outfit. As in Phase 2 of Study 2, participants estimated the final mark and thesis points and rated students’ sexiness (α ranging from 0.83 to 0.92), competence (α ranging from 0.80 to 0.91), and outfit appropriateness. All answers were provided on a scale from 1 (*not at all*) to 7 (*completely*). Participants filled out the OBCS (α = 0.76) and the objectifying TV consumption scale (α = 0.80) as in previous studies. Finally, before being thanked and debriefed, they indicated whether they knew any of the persons portrayed in the photos.

#### Materials

##### Photos

Twenty-four pictures were selected from those used in Study 2. Twelve of these pictures involved sexy outfits consisting in short dresses or skirts, evident necklines and high heels, whereas 12 pictures involved a professional outfit with jacket and trousers. The two types of outfits had been judged differently by raters of Study 2. Professional outfits were judged as more masculine (*M* = 3.17, *SD* = 0.98) but less sexy (*M* = 3.23, *SD* = 0.70) than the sexy outfits [*M*_masculine_ = 1.62, *SD* = 0.62; *t*(22) = 4.60, *p* < 0.001 and *M*_sexy_ = 4.31, *SD* = 0.63; *t*(22) = −3.99, *p* = 0.001].

### Results

All dependent variables were submitted to a 2 (outfit: sexy vs. professional) × 2 (participant gender: male vs. female) ANOVA where the first was a within- and the second a between-participants variable. As shown in Table 2, confirming Hypothesis 1, participants attributed more thesis points and a higher final mark to the students wearing a professional (vs. sexy) outfit and, supporting Hypothesis 2, students dressed professionally were perceived as more competent and less sexy than those dressed sexy. Finally, as predicted by Hypothesis 3, they perceived the professional outfit as more appropriate for the occasion than the sexy one.

**Table 2:** Means and standard deviation, and main effects of outfit on all the dependent variables (Study 3).

	Sexy *M* (*SD*)	Professional *M* (*SD*)	*F*-values
Thesis points	6.63 (1.20)	7.36 (0.92)	*F*(1,71) = 34.94, *p* < 0.001, 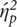 = 0.33
Final mark	97.00 (8.43)	100.68 (7.29)	*F*(1,71) = 4.76, *p* = 0.03, 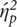 = 0.06
Competence	4.09 (0.91)	4.87 (0.84)	*F*(1,71) = 58.74, *p* < 0.001, 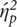 = 0.45
Sexiness	3.80 (1.27)	3.22 (1.04)	*F*(1,71) = 17.64, *p* < 0.001, 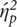 = 0.20
Outfit appropriateness	3.55 (1.25)	4.99 (1.17)	*F*(1,71) = 58.50, *p* < 0.001, 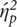 = 0.45

Some of these effects were qualified by significant interactions with participants’ gender (Table 3). In particular, male participants tended to judge the students dressed sexy as more sexy, but also as more competent and appropriate than female participants did. No gender differences were instead found in the perception of professionally dressed students.

**Table 3:** Outfit by Gender interaction on all the dependent variables (Study 3).

	Sexy *M* (*SD*)		Professional *M* (*SD*)		*F*-values
	Male	Female	Male	Female	
Thesis points	6.77 (1.12)	6.50 (1.28)	7.28 (0.94)	7.43 (0.91)	*F*(1,71) = 2.96, *p* = 0.09, 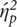 = 0.04
Final mark	98.59 (6.77)	95.45 (9.62)	101.02 (7.41)	100.35 (7.26)	*F*(1,71) = 2.34, *p* = 0.13, 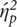 = 0.03
Competence	4.36 (0.83)^a^	3.83 (0.93)^b^	4.94 (0.84)^c^	4.81 (0.85)^c^	*F*(1,71) = 3.75, *p* = 0.06, 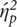 = 0.05
Sexiness	4.36 (1.16)^a^	3.25 (1.14)^b^	3.14 (1.12)^c^	3.30 (0.95)^c^	*F*(1,71) = 20.27, *p* < 0.001, 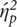 = 0.22
Outfit appropriateness	3.99 (1.14)^a^	3.12 (1.22)^b^	5.00 (1.20)^c^	4.98 (1.16)^c^	*F*(1,71) = 5.11, *p* = 0.03, 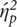 = 0.07

*When a significant interaction was found, pairwise comparisons (Bonferroni correction) were performed and, for each dependent variable, means were associated with a superscript. Means with different subscripts are significantly different from each other at p < 0.05. For instance, the mean with superscript “a” was significantly different from the means with superscript “b” and “c” reported on the same row. When two means have the same superscript, they were not statistically different from each other. No subscripts are reported for non-significant interaction effects.*

#### Mediation Analyses

As in Study 1, we explored whether outfit impacted the perceived competence, which, in turn, affected the estimation of students’ graduation outcome. As Study 2 showed the importance of outfit appropriateness, in this case we tested the indirect pathways from outfit appropriateness to final mark and thesis point via perceived competence by using Hayes’ (2013) PROCESS macro (model 4) and bias-corrected intervals (5000 bootstrap resamples). Due to the within-participants design of the study we first calculated an index for each variable by subtracting ratings for sexy dressed students from those of professionally dressed students. Hence, higher scores indicate that students dressed professionally (vs. sexy) were attributed higher final marks, more thesis points, and greater competence and that their outfit was perceived as more appropriate compared to the sexy students. Analyses showed that the effect of outfit appropriateness on thesis points (*b* = 0.24, *SE* = 0.07, *t* = 3.40, *p* = 0.001) became non-significant when competence was introduced in the model as mediator (*b* = 0.02, *SE* = 0.07, *t* = 0.16, *p* = 0.86). The indirect effect (*b* = 0.23, *SE* = 0.06, 95% CI [0.12, 0.38]) as well as the Sobel test (*z* = 4.02, *p* < 0.001) were significant. The same analysis performed on final mark showed the same pattern of results. The effect of outfit appropriateness on final mark was mediated by perceived competence (*b* = −0.13, *SE* = 0.48, *t* = −0.27, *p* = 0.78) as shown by the indirect effect (*b* = 1.52, *SE* = 0.50, 95% CI [0.71, 2.77]) and Sobel test (*z* = 3.98, *p* < 0.001).

#### Objectifying TV Consumption and Self-Objectification

We performed linear regression analyses to test whether objectifying TV consumption (*M* = −0.05, *SD* = 0.41) and self-objectification (*M* = 3.31, *SD* = 0.83) predicted observers’ judgments. None of the models was found to be significant (*R*^2^ < 0.07, *F*s < 0.98, *p*s > 0.38) indicating that these individual differences did not explain participants’ judgments.

### Discussion

Study 3 replicated previous findings by showing that professionally dressed students who recently graduated were perceived as more competent, less sexy, and more appropriate to the context than those dressed in a sexy attire. Moreover, extending results of Study 1 and Study 2, our results showed that outfit appropriateness affected the perception of competence, which, in turn, led observers to estimate the final mark and thesis points obtained by the students. It seems that wearing an outfit considered as appropriate increases the perception of the students’ competence and ultimately the expectation that they will be successful in getting a higher mark.

Surprisingly, in this study we found self-objectification and objectifying TV consumption not to influence observers’ ratings. This lack of effects is in contrast with findings of Study 1 and may be explained by differences in designs, sample size, and participants’ age.

## General Discussion

This work has examined for the first time how graduation outfits are chosen by female students and how their attire affects observer judgments. Overall, female students chose their graduation outfit in order to appear beautiful and to express their personality and less so to appear competent. Hence, appearing competent seemed not to be their main concern. At the same time, outfit had a reliable impact on observers who attributed less effort and poorer outcomes (thesis points and higher final mark) to the students dressed in a sexy fashion (Study 1 and Study 3). Sexy outfits were even associated with fewer thesis points actually obtained by students in real life (Study 2). This result suggests that graduation committees may have been influenced by the students’ attire when evaluating their theses. Very similar patterns of results were found both when exposing participants to experimentally manipulated pictures (Study 1) or to graduation outfits worn in real life (Studies 2 and 3).

This research contributes to the literature on women’s clothing and sexualization in several respects. First, it shows that the outfit students decide to wear for their graduation can have tangible consequences. Sexy dresses are not only considered inappropriate for this specific occasion (graduation), but they also reflect negatively on the student and affect the (real and imagined) performance negatively. Interestingly, although students and professors usually have divergent opinions on clothing (Ruetzler et al., 2012), here we found young female students, adults and professors to show similar attitudes (see Cabras et al., 2018).

Second, extending previous research (Glick et al., 2005; Howlett et al., 2015), we investigated the underlying process, finding that outfit impacts the perception of students’ competence, which in turn affects the estimated grade. Moreover, it is not only the sexiness of the outfit, but also its lower appropriateness, that determines the lower perceived competence and decline in performance.

Third, it shows that young women have to deal with the pressure of choosing an outfit that is considered appropriate to the event, matches observers’ expectations (Smolak et al., 2014), and also fits their personal goals (e.g., appearing beautiful and expressing the self). To juggle these different demands may require remarkable acrobatic skills.

### Limitations and Future Research

We examined here the role of “sexy” and “professional” outfits as if they were, by definition, opposite ends of the same spectrum. Although this is in line with much of the prior literature (e.g., Glick et al., 2005), sexiness and professionalism may not necessarily be mutually exclusive. Future studies should examine whether sexy and non-sexy professional outfits may elicit similar first impressions.

Moreover, this research aimed at exploring individual and societal characteristics that may moderate observers’ judgments. We did find effects of the attention people pay to their own appearance on how they evaluate others. This is particularly interesting as the majority of prior research had focused on what increases self-objectification and how self-objectification affects personal behaviors (Calogero et al., 2005), with few exceptions showing that higher self-objectification was linked to objectification of other women (Strelan and Hargreaves, 2005). Here, we showed that observers low in self-objectification judged sexy (vs. professionally) dressed students more negatively. In contrast, strongly self-objectifying participants did not show this bias indicating that they evaluated professionally and sexy dressed students similarly, presumably due to the fact that they perceive objectifying self-presentations more normative. This is not surprising given that people tend to project their own behaviors, opinions and preferences onto others (false consensus bias; Marks and Miller, 1987). However, since in our samples self-objectification was overall low, future studies should extend our research by considering more representative samples and different contexts.

We also examined whether objectifying TV consumption would have consequences for how individuals evaluate others from what they wear. Although not entirely consistent across studies, the exposure to objectifying TV seems to produce some effects. Study 1 showed that people consuming more objectifying TV were less affected by the type of outfit students wore. This seems to suggest that exposure to objectifying TV may change people’s beliefs about what is normative (Ward, 2016). If sexualized representations of women become the norm, then students dressed sexy for their graduation may be seen positively as they match the standards set by mass media. However, our research assessed the overall frequency of watching objectifying TV shows without differentiating between types of show (e.g., comic, reality shows, dating shows, etc.) and without considering the level of viewer’s “awareness” or media literacy. It is conceivable that some TV consumers watch objectifying television because of their entertainment value, while still maintaining a critical perspective on how women are portrayed. This is an interesting and, to our knowledge, under-investigated question. Testing whether individuals are critical watchers, rather than passive “consumers” of such TV programs, may be a more effective way of examining the role of television on individuals’ attitudes toward women and their clothes. Also, our findings indicated that our students’ outfit choices were not related to their objectifying TV consumption. TV offers ideal images of women but, currently, young women may be more likely to consult internet and social networks to decide what to wear, even for their graduation. Hence, future research should explore the role of various types of mass media in influencing clothing choices for formal occasions such as graduation (Fardouly et al., 2015) as well as compare social contexts and countries that vary in exposure and frequency of objectifying TV consumption.

Another aspect that could be taken in consideration in future research is the role of specific types of garments on observer perception. Our research focused on the whole outfit, but research has shown that single elements like shoes (Gillath et al., 2012), or make up may play specific roles in first impressions (Ruetzler et al., 2012). Moreover, research on enclothed cognition suggests that clothes are connoted with symbolic meanings that affect not only the observer’s impressions but also the wearers’ performance (Adam and Galinsky, 2012). It would therefore be important to investigate the meaning associated with specific graduation outfits and how wearing a sexy (vs. a professional) outfit affects the students’ performance in defending their thesis (Tiggemann and Andrew, 2012b).

Also, this research should be extended to male students. In this case, unprofessional graduation outfits are more likely to be those perceived as more casual and/or as less masculine rather than sexy. Finally, future research should pay closer attention to cultural contexts, including both differences between nations and between fields (e.g., Arts vs. Sciences) as different cultures are likely to embrace distinct implicit clothing norms. This study was conducted in Italy, a Western country where attention to fashion is common, and on samples that consisted of Catholic and politically liberal participants. Since political and religious beliefs play a role on clothing preference (Buckley and Roach, 1974; Edmonds and Cahoon, 1993), future research should extend these findings to more diverse contexts and samples.

### Practical Implications

Our work examined a context, namely school/university, that has been a target of debate about clothing appropriateness. Schools and universities have repeatedly condemned certain outfits and labeled them as inappropriate for the context, in response women have claimed the right to dress as they wish without being penalized. Our findings suggest that there exists a shared idea that certain clothes are preferred to others in educational contexts and that, if female students do not conform to such clothing expectations/norms, they may be target of negative evaluations. In particular, we are witnessing a paradoxical situation for women. They are increasingly pushed by media and peers to sexualize their appearance in order to feel attractive and competent in professional contexts. Nevertheless, once their appearance is sexualized, they reach the opposite result as they tend to be perceived as *less* competent. Thus, interventions are needed to increase awareness among young women concerning the potential negative consequences of self-sexualization for academic performance. More importantly, observers and, specifically professors, should be made aware of the outfit bias in order to guarantee objective performance evaluations regardless of how the students are dressed (Johnson and Gurung, 2011).

Our findings indeed indicate that, although people generally agree that they should not “judge a book by its cover,” they still do so even in the context of higher education where intellectual performance, and not the student outfit, should be valued.

## Ethics Statement

This research was carried out in accordance with the recommendation of the Ethic committee of Dipartimento di Psicologia dello Sviluppo e della Socializzazione (University of Padua). The protocol was approved by the Ethic committee of Dipartimento di Psicologia dello Sviluppo e della Socializzazione (University of Padua). All participants read the participants information sheet and provided written informed consent in accordance with the Declaration of Helsinki.

## Author Contributions

FF, AM, CV, and MGP: conceptualization and design of the research. FF: data collection. FF and AM: data analyses and writing – original draft. FF, AM, CV, and MGP: writing – review and editing and final approval of the version to be published.

## Conflict of Interest Statement

The authors declare that the research was conducted in the absence of any commercial or financial relationships that could be construed as a potential conflict of interest.
