# Supplementary material for: The (Female) Graduate: Choice and Consequences of Women’s Clothing
Source: Front Psychol. 2018 Nov 29;9:2401. doi: 10.3389/fpsyg.2018.02401 (PMC6281884; doi:10.3389/fpsyg.2018.02401)
Supplement: Supplementary file 1 [file Table_1.DOCX]

**Supplementary Information – SI 1**

The (female) graduate: Choice and consequences of women’s clothing

***Photographed Students’ survey***

Photographed students completed a survey in which they were asked to indicate which of the two outfits better represented their personality, competence, beauty and which one they would choose for their own thesis defense. Answers were provided on a scale from -3 (surely the professional outfit) and 3 (surely the sexy outfit), with 0 indicating no preference for either outfit. Our volunteers also indicated how important they personally considered competence and beauty, and responded to a self-objectification measure (Objectified Body Consciousness Scale; McKinley & Hyde, 1996).

Our female volunteers indicated that the professional outfit was expressing their competence (*M* = -2.05, *SD* = 1.22) and personality (*M* = -1.17, *SD* = 1.69) more than the sexy one (*t*-test against the mid-point: *t*s < -4.21, *p*s < .001), whereas the sexy outfit was judged to express their beauty better (*M* = .94, *SD* = 1.20, *t*(36) = 4.75, *p* < .001, *d* = 1.58). Participants reported that they would have chosen the professional over the sexy outfit (*M* = -1.65, *SD* = 1.65, *t*(36) = -6.06, *p* < .001, *d* = 2.02), but responses varied considerably across participants (76% opted for the professional outfit, 8% for the sexy outfit, whereas 16% were undecided). To test whether the choice of the graduation outfit was predicted by self-objectification, and by the importance of competence over beauty – an index calculated by subtracting ratings for importance of beauty from those of competence – we ran a regression analysis in which these two measures were used as predictors and the choice of the outfit as dependent variable (all z-transformed). Self-objectification emerged as the only reliable predictor of outfit choice, *β* = .45, *t* = 2.86, *p* = .007 (for all correlations see Table below).

Table. *Means and Standard Deviations, and correlation between female students’ ratings on outfit judgments and self-presentation.*

|  | *M* | *SD* | 1 | 2 | 3 | 4 | 5 | 6 | 7 |
| --- | --- | --- | --- | --- | --- | --- | --- | --- | --- |
| 1. Outfit competence | -2.05 | 1.22 | 1 |  |  |  |  |  |  |
| 2. Outfit beauty | .94 | 1.20 | .23 | 1 |  |  |  |  |  |
| 3. Outfit personality | -1.17 | 1.69 | .50** | .04 | 1 |  |  |  |  |
| 4. Outfit choice | -1.65 | 1.65 | .38** | .40* | .35* | 1 |  |  |  |
| 5. Importance for competence | 4.36 | .77 | .18 | .18 | .12 | .27 | 1 |  |  |
| 6. Importance for beauty | 3.55 | .64 | .17 | .34* | .26 | .32 | .42** | 1 |  |
| 7. Self-objectification | 3.07 | .48 | .41* | -.00 | .22 | .50** | .22 | .17 | 1 |

**Supplementary Information – SI 2**

***Answers to the open-ended question – Study 2 – Phase 1***

In Phase 1 of Study2, participants answered an open-ended question specifying how and why they had chosen that particular outfit. Answers to this question were coded. Participants referred to four main content areas (i.e., personality and feeling at ease, beauty, professionalism, and appropriateness) and the number of references to each category was counted. The majority of participants mentioned at least once the desire of appearing beautiful and elegant (52.7% of respondents, n = 60), some students reported at least once to have chosen their outfit in order to express their personality and feeling at ease (32.5%, n = 37), to appear professional (29.8%, n = 34), and/or appropriate to the context (26.3%, n = 30). This information, derived from phase 1, was taken into consideration when developing the measures of the following phase of the study.
